# The impact of social isolation on smartphone addiction among college students: the multiple mediating effects of loneliness and COVID-19 anxiety

**DOI:** 10.3389/fpsyg.2024.1391415

**Published:** 2024-07-22

**Authors:** Ye Wang, Qianying Ma

**Affiliations:** ^1^School of Journalism & Communication, Jinan University, Guangzhou, Guangdong, China; ^2^School of Public Administration, Nanfang College Guangzhou, Guangzhou, Guangdong, China; ^3^Faculty of Modern Languages and Communication, Universiti Putra Malaysia, Seri Kembangan, Selangor, Malaysia

**Keywords:** social isolation, loneliness, COVID-19 anxiety, smartphone addiction, multiple mediations

## Abstract

**Background:**

The widespread use of smartphones has significantly increased smartphone addiction among college students, especially during the COVID-19 pandemic. Since the COVID-19 outbreak, university campuses in mainland China have strictly followed the Chinese government’s strict quarantine policy, including closed campus management, prohibitions against gatherings, and social distancing, increasing student loneliness and anxiety and thus increasing the risk of smartphone addiction. Extant Studies have revealed that social isolation is a prominent predictor of smartphone addiction but have failed to systematically explore the complex relationship between social isolation and smartphone addiction in the context of COVID-19; therefore, the underlying mechanisms of these factors in the post-pandemic era are unclear.

**Objective:**

This study is the first attempt to consider loneliness, COVID-19 anxiety and social isolation as a whole and to clarify the underlying mechanisms of social isolation and smartphone addiction by constructing a multiple mediating model.

**Method:**

This study included students enrolled at eight higher education institutions in Conghua District (Guangzhou city). Quota proportional sampling was adopted, 900 self-report questionnaires were distributed through the WeChat groups of these universities from December 10 to December 15, 2022, and 868 valid questionnaires (620 females, 248 males) were ultimately obtained.

**Results:**

The direct effect of social isolation on smartphone addiction was significant, and loneliness and COVID-19 anxiety mediated the association between social isolation and smartphone addiction in both parallel and sequential ways. Moreover, a reverse mediation model with COVID-19 anxiety as the first mediator and loneliness as the second mediator was found.

**Conclusion:**

College students who experience social isolation are at greater risk for smartphone addiction, and the core factor leading to their smartphone addiction is the subjective psychological state triggered by campus isolation and social distancing, such as loneliness and anxiety. These risky behaviors of people should receive extra attention, and psychological factors such as loneliness and COVID-19 anxiety should be considered in future therapies aimed at reducing addiction.

## 1 Introduction

In the last two to three decades, smartphones have emerged as crucial instruments for daily human activities. By June 2023, the number of mobile Internet users in China had increased to 1.076 billion. A total of 99.8% of the participants accessed the Internet through mobile devices, and the average weekly Internet usage time of Chinese users reached 29.1 h (China Internet Network Information Center, 2023). However, excessive use of smartphones may seriously interfere with individuals’ lives and learning, leading to psychological and behavioral disorders of daily functioning (Cheever et al., 2014), for example, smartphone addiction (Chiu, 2014). Smartphone addiction arises when individuals cannot control their usage, resulting in psychological and behavioral disturbances that impair daily functioning (Liu et al., 2017). Numerous studies have shown that college students are heavy users of smartphones and are more prone to impulsivity and a lack of self-control than other groups, thus increasing their susceptibility to smartphone addiction (Liu et al., 2020); therefore, this study focused on college students.

Notably, smartphone addiction among college students was particularly pronounced during the COVID-19 pandemic (Hosen et al., 2021; Albursan et al., 2022; Hu et al., 2022). At the end of 2022, COVID-19 reemerged in mainland China, and strict closed-campus management measures were implemented on university campuses as key sites for epidemic prevention, including closed campus management, restricted student mobility, demarcated study and living areas, avoidance of gatherings, social distancing in public places, and banning of outside visitors to dormitories. These measures were accompanied by frequent infections, which seriously interfered with regular teaching activities and students’ academic development and undoubtedly increased the possibility of developing smartphone addiction (Hosen et al., 2021; Santander-Hernández et al., 2022; Wu et al., 2022). Smartphone addiction can cause numerous adverse effects on college students, such as physical health problems (Zhang and Wu, 2020), sleep disorders (Haripriya et al., 2019), academic failure (Albursan et al., 2022), depression (Demirci et al., 2015), and social anxiety (Caponnetto et al., 2021; Zhan et al., 2021). Therefore, exploring the antecedents and underlying mechanisms of smartphone addiction among college students in the post-pandemic period is particularly important for formulating prevention and intervention strategies for smartphone addiction among college students.

Extant empirical studies have demonstrated that social isolation is a significant antecedent to smartphone addiction. Social isolation greatly limits the range of activities and socialization of college students, and smartphones have become their primary means of communication, socialization, and entertainment, playing a crucial role in de-escalating feelings of loneliness and anxiety and providing an important channel for accessing COVID-19-related information (Hosen et al., 2021). Through smartphones, college students can stay connected to the outside world and stay up-to-date on the epidemic, which also amplifies the risk of cell phone addiction. Previous studies have focused on exploring the characteristics and influences of smartphone addiction, including age (Hallauer et al., 2021; Wen et al., 2023), interpersonal relationships (Zhen et al., 2019), self-esteem (Kim and Koh, 2018), life stress (Chiu, 2014), and smartphone use (Zhao and Zhou, 2021), in individuals during the COVID-19 pandemic. However, systematic research on the relationship between social isolation and cell phone addiction during the COVID-19 pandemic is still insufficient, and although some studies have attempted to explore this relationship (e.g., Al-Kandari and Al-Sejari, 2021; Zhen et al., 2023), there is a lack of dedicated research on the direct links and potential mediating processes between the two.

In contrast to previous pathological and compulsive perspectives (e.g., Billieux et al., 2015; Choi et al., 2015), compensatory internet use theory (CIU) reveals the importance of interpreting cell phone addiction from a compensatory perspective. When people experience problems in the real world, they may turn to smartphones to escape pain (Kardefelt-Winther, 2014). People with high levels of loneliness and anxiety use smartphones for social interaction, online entertainment and recreation to alleviate the pain of loneliness and anxiety, and they can easily develop smartphone addiction (Kim et al., 2017; Shen and Wang, 2019). The extensive use of lockdown, quarantine, and social distancing tactics during the COVID-19 pandemic highlights the need to research social isolation and mobile phone addiction from the standpoint of psychological compensation (Kardefelt-Winther, 2014). Scholars have individually discussed how social isolation (Al-Kandari and Al-Sejari, 2021; Zhen et al., 2023), loneliness (Tan et al., 2013; Shen and Wang, 2019; Zhen et al., 2019), and anxiety (Kim and Koh, 2018; Elhai et al., 2019; Liu et al., 2020) affect smartphone addiction. However, the various factors have not been considered as a whole. As a result, it is unclear whether social isolation, loneliness, and anxiety have combined effects on smartphone addiction.

The present study attempts to fill these research gaps by constructing a multiple mediation model based on CIU theory by incorporating loneliness and COVID-19 anxiety as mediating variables to reveal the multiple mechanisms underlying the relationship between social isolation and smartphone addiction. This study will be an important supplement to the literature on the antecedents of smartphone addiction in the context of public crises and will provide recommendations for the intervention and prevention of smartphone addiction among college students during future pandemics.

### 1.1 Social isolation and smartphone addiction

Smartphone addiction is often labeled “smartphone overuse,” “problematic smartphone use,” or “smartphone dependence,” but these terms refer to different constructs. Smartphone addiction is characterized as a nonsubstance-related addiction that emerges from the excessive utilization of various functionalities of smartphones. This form of addiction adversely affects individuals’ psychological, physiological, and social well-being (De-Sola Gutiérrez et al., 2016). Individuals experiencing smartphone addiction typically exhibit extensive usage time and a lack of control over their usage frequency. Such addictive behaviors detrimentally influence individuals’ learning processes and daily life activities and impair their social functionalities (Liu et al., 2020).

Being physically, psychologically, or in any combination of these ways cut off from desired interpersonal relationships is known as social isolation (Nicholson, 2012). This phenomenon can be quantitatively assessed as an objective state, indicated by factors such as living in solitude, having limited social contact, and engaging in minimal social activities (Dahlberg, 2021). Additionally, social isolation can manifest as a subjective experience that may include feelings of not belonging to a group (Nicholson et al., 2020) or the perceived disparity between actual and desired social interactions (Hajek and König, 2019). Previous studies have often viewed social isolation as a one-dimensional variable, while this study views it as multidimensional and as a complex of objective states and subjective feelings.

Research on the psychology of smartphone addiction is very rich, and researchers have explored the effects of depression, anxiety, stress, and self-esteem on smartphone addiction. Social isolation is a prominent antecedent factor contributing to smartphone addiction during the COVID-19 pandemic (Zhen et al., 2023). A considerable volume of empirical research has established a correlation between social isolation and smartphone addiction (e.g., Kim and Jung, 2021; Volpe et al., 2022; Zhen et al., 2023). A notable example is a study involving 1,431 young individuals aged 17 to 26 years in Kuwait that found that an increased degree of social isolation corresponded with a heightened level of smartphone addiction. In this study, social isolation emerged as a significant predictor of smartphone addiction (Al-Kandari and Al-Sejari, 2021).

Additionally, research conducted during the COVID-19 pandemic has drawn similar conclusions. A cross-national study of older populations in 62 countries (Kim and Jung, 2021) and a longitudinal survey of Chinese college students (Hu et al., 2022) demonstrated that social isolation can lead to smartphone addiction.

Based on these findings, this study proposes the following hypotheses:

*H1:* Social isolation is positively related to smartphone addiction.

### 1.2 The mediating role of loneliness

The experience of loneliness stems from a perceived gap between the social connections one desires and those one actually has, often emerging when there is a perceived deficiency in either the quality or quantity of one’s social ties (Perlman and Peplau, 1981). This psychological condition manifests as a profound feeling of void, perceived worthlessness, diminished autonomy, and an increased sense of vulnerability (Cacioppo and Patrick, 2008).

Empirical research has demonstrated that the association between social isolation and smartphone addiction is mediated by loneliness (e.g., Zhen et al., 2023). On the one hand, social isolation may increase loneliness. Although isolation is an effective measure against the spread of COVID-19 (Herrera-Diestra and Meyers, 2019), prolonged social isolation, especially when mandatory, may weaken social interactions and thus trigger loneliness (Wilkialis et al., 2021). According to Maslow’s hierarchy of needs, social needs are a fundamental human requirement (Maslow, 1943). The extension of social isolation, particularly under nonnatural conditions (such as in response to COVID-19), may lead to increased loneliness (Brooks et al., 2020; Dahlberg et al., 2022). A systematic literature review based on 24 papers from 10 countries showed that isolation measures taken during global infectious disease (such as SARS, Ebola, and H1N1 influenza) all lead to increased feelings of loneliness (Salazar De Pablo et al., 2020). On the other hand, loneliness may lead to smartphone addiction. The most likely and convenient way for college students to escape and alleviate the feelings of loneliness induced by social isolation from offline activities and gatherings is access to the Internet via smartphones during the COVID-19 pandemic (Kim et al., 2015). The greater the feeling of loneliness is, the greater the likelihood of smartphone addiction (Liu et al., 2020; Santander-Hernández et al., 2022).

Therefore, the following research hypotheses are proposed:

*H2:* Social isolation is positively related to loneliness.

*H3:* Loneliness is positively related to smartphone addiction.

*H4:* Loneliness mediates the relationship between social isolation and smartphone addiction.

### 1.3 The mediating role of COVID-19 anxiety

Uncertainty about the future course and duration of COVID-19 has triggered widespread anxiety, fear, uncertainty, and insecurity (Ahorsu et al., 2022). COVID-19 anxiety is caused by the emotions of apprehension, fear, and worry provoked by COVID-19 (Yıldırım et al., 2022). This anxiety includes not only the fear of contracting or being reinfected with COVID-19 but also concerns about its potential future impacts, such as economic and health impacts, as well as challenges in terms of unemployment and relationships (Tull et al., 2020).

Many empirical studies have confirmed that public anxiety increases during major infectious disease outbreaks, such as 2003 SARS and 2014 MERS (Huang and Zhao, 2020; Salazar De Pablo et al., 2020). One study noted that the extended strict isolation policy associated with the COVID-19 pandemic greatly increased anxiety levels among college students during the lockdown (Li et al., 2023). A systematic literature review exploring anxiety and smartphone addiction showed that anxiety severity was consistently and significantly associated with smartphone addiction (Elhai et al., 2017). A positive correlation between the two was also confirmed in the context of COVID-19 (Al Qudah et al., 2021; Kim et al., 2021). People often resort to smartphones to distract themselves from negative emotions. For example, people experiencing anxiety may seek psychological comfort by relying on their smartphones for regular updates and communication (e.g., phone calls, text messages) or by engaging in recreational activities to alleviate anxiety (Lopez-Fernandez et al., 2018), which may inadvertently lead to addiction (Busch and McCarthy, 2021; Hou et al., 2023). Based on these findings, the following hypotheses are proposed:

*H5:* Social isolation is positively related to COVID-19 anxiety.

*H6:* COVID-19 anxiety is positively related to smartphone addiction.

*H7:* COVID-19 anxiety mediates the relationship between social isolation and smartphone addiction.

### 1.4 The multiple mediation model

Mediating effects may be parallel, sequential, or mixed (Hayes, 2012). As mentioned in the previous hypothesis, loneliness and COVID-19 anxiety may be parallel mediators of the relationship between social isolation and smartphone addiction. Furthermore, while direct evidence has not been extensively documented, certain studies indirectly imply that loneliness and COVID-19 anxiety may exert a sequential mediating influence. The effects of loneliness on mental health are substantial and often regarded as precursors to various psychological and physical health issues, including depression, anxiety, cognitive decline, and physical ailments. The lockdown measures taken during the COVID-19 pandemic led to heightened social isolation and have been associated with increased loneliness due to reduced interpersonal interactions (Leal Filho et al., 2021). Individuals experiencing loneliness are more vulnerable to a spectrum of adverse psychological health consequences, such as anxiety and depression (Palgi et al., 2020; Wu et al., 2021). Additionally, existing research underscores anxiety as a contributing factor to the rise in smartphone addiction (Visweswaraiah et al., 2021; Santander-Hernández et al., 2022). Based on these findings, the following hypotheses are proposed:

*H8:* Loneliness is positively related to COVID-19 anxiety.

*H9:* Loneliness and COVID-19 anxiety mediate the relationship between social isolation and smartphone addiction in both parallel and sequential way.

**Figure 1:**
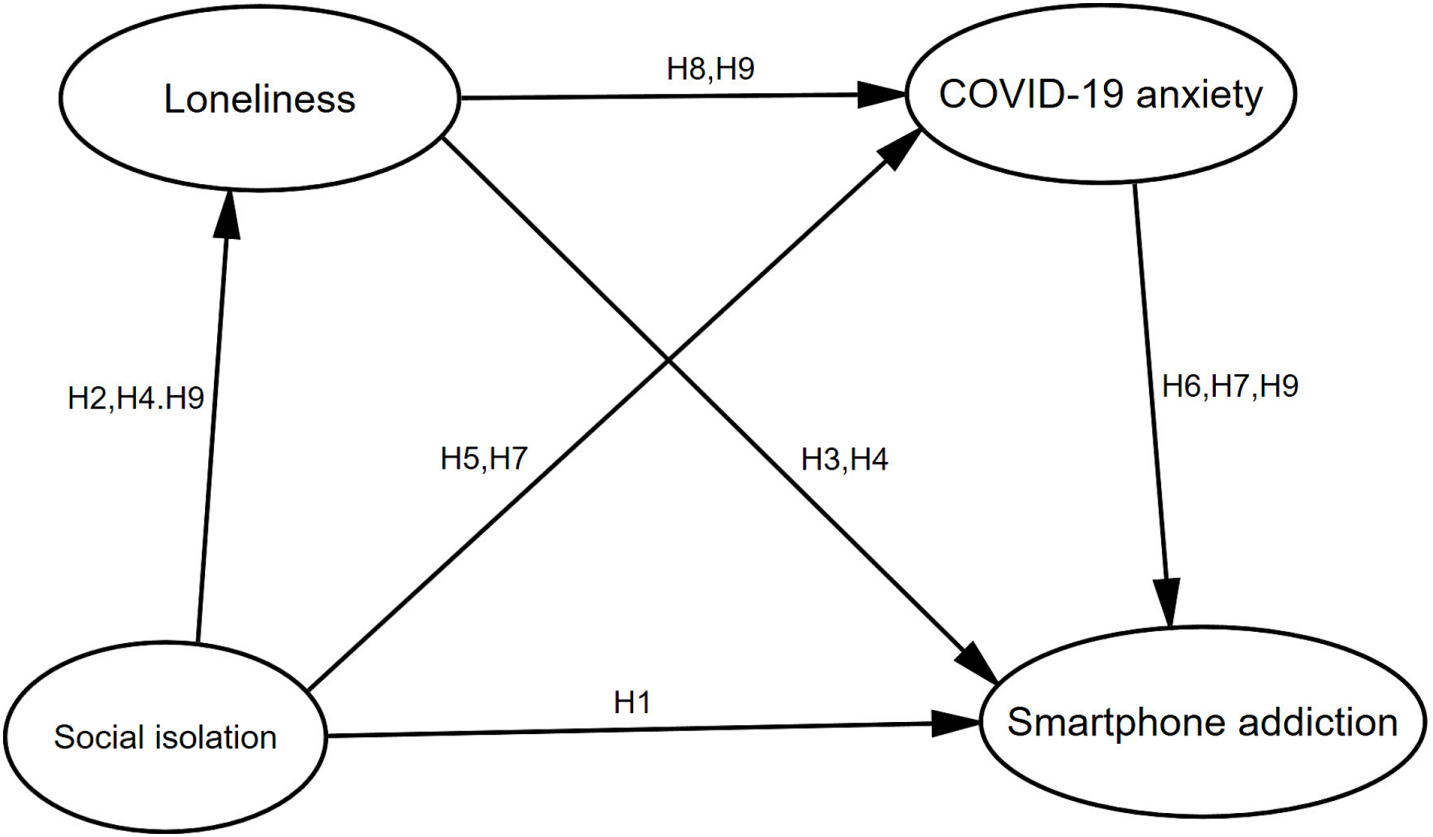
Conceptual framework.

In conclusion, the present study aims to investigate the underlying associations between social isolation, loneliness, COVID-19 anxiety, and smartphone addiction. Specifically, we examined the multiple mediation model (Figure 1), outlined based on the aforementioned hypotheses to address four key questions: (a) whether social isolation can predict college students’ smartphone addiction; (b) whether loneliness mediates the relationship between social isolation and smartphone addiction; (c) whether COVID-19 anxiety mediates the relationship between social isolation and smartphone addiction; and (d) whether these two variables operate in both parallel and sequential way.

## 2 Method

### 2.1 Participants and procedures

This study included students enrolled at eight higher education institutions in Conghua District, Guangzhou city. Conghua is located in the northeastern part of Guangzhou city and is an important center of higher education in Guangdong Province. At the end of 2022, COVID-19 erupted again in Guangzhou, and Conghua was one of the hardest hit areas, with colleges and universities in the district implementing stricter closed-campus policies. In addition, the district is located in a suburb and is not easily accessible, which may have exacerbated students’ feelings of isolation and anxiety and provided an excellent sample for the study.

A cross-sectional survey method was used, with quota sampling based on the proportion of students enrolled at eight higher education institutions in Conghua District (Table 1). A link to the questionnaire was generated through the Questionnaire Star platform and shared with current students through the WeChat groups of each university. The questionnaire’s preamble section provided a brief overview of the study’s main purpose, assuring respondents that their answers would remain confidential and not be disclosed to any third party. Respondents can choose to agree or directly withdraw from the questionnaire. Choosing “agree” allows them to continue answering the questionnaire. The participants took approximately 3 min to complete the online questionnaire. Questionnaire collection was stopped once a predetermined number of questionnaires was reached at each university. Between December 10, 2022, and December 15, 2022, 900 samples were collected. After removing invalid questionnaires with overly short completion times (less than 100 s) and those with more than two-thirds of the questions having the same answers, a total of 868 valid samples were retained.

**Table 1:** Quota sampling method.

College	Number of enrolled students^a^	Questionnaire distribution ratio	No. of questionnaires distributed	No. of valid questionnaires collected
Guangdong Engineering Polytechnic	11,000	9.57%	86	80
Guangzhou Medical College Institute of Conghua	7,800	6.79%	61	60
Nanfang College · Guangzhou	20,127	17.51%	158	158
Guangdong Polytechnic of Water Resources and Electric Engineering	18,000	15.66%	141	141
Zhujiang College of South China Agricultural University	11,000	9.57%	86	80
Software Engineering Institute of Guangzhou	15,000	13.05%	117	102
Guangzhou Nanyang Polytechnic College	12,000	10.44%	94	92
Guangzhou City Construction College	20,000	17.40%	157	155
Total	222,927	100%	900	868

a Enrolled students include undergraduates, higher vocational college students, and adult education students (updated September 30, 2022).

### 2.2 Measures

This study designed a questionnaire with a total of 29 items. The questionnaire was divided into five sections: demographic background, social isolation scale, loneliness scale, COVID-19 anxiety scale, and smartphone addiction scale. The demographic background information collected pertained to gender, age, college and grade level attended, and monthly living expenses.

#### 2.2.1 Social isolation

The scale developed by Nicholson et al. (2020) was applied because it fully accounts for important developments in technology that may affect social isolation (e.g., e-mail, video chatting, and the Internet). This scale includes two dimensions: objective states and subjective feelings. It consists of six items. Three items, such as “you do not have a sense belonging” and “You feel you did not spend enough time in social activities,” were used to test the participants’ connections to the group, which are subjective feelings, with a 5-point Likert scale (1 = never to 5 = always). The other three items asked participants to answer a number of questions: (1) How many of your family members, friends, and neighbors do you see at least once a month? (2) How many of your relatives, friends, and neighbors do you communicate with at least once a month by phone or electronically (e.g., email, video chat) via the Internet? and (3) How many of your relatives, friends, and neighbors do you feel close to on a personal level (e.g., can you confide in them or share personal feelings?). These questions measured objective states with a 5-point Likert scale (1 = “6 or more,” 2 = “4–5,” 3 = “2–3,” 4 = “1,” 5 = “None”).

#### 2.2.2 Loneliness

Loneliness was measured according to the Hughes et al. (2008) scale, which has been extensively applied within pandemic research contexts. This scale includes items such as “How often do you feel you lack companionship?” Scoring is based on a 5-point Likert scale (1 = never, 5 = always), where elevated scores signify increased loneliness.

#### 2.2.3 COVID-19 anxiety

Adopted from Yıldırım et al. (2022), five items were used to measure respondents’ feelings of apprehension, fear, or foreboding due to COVID-19. Participants were asked whether (1) they were anxious about their future, (2) they were worried about their health, (3) they were anxious about contracting (or recontacting) COVID-19, (4) they were preoccupied because they were worried about contracting COVID-19, and (5) they were afraid of COVID-19 affecting their lives. The scores are based on a 5-point Likert scale (1 = strongly disagree to 5 = strongly agree), where elevated scores signify increased COVID-19 anxiety.

#### 2.2.4 Smartphone addiction

The smartphone addiction scale (Kwon et al., 2013) was employed to measure this construct, comprising ten items including “missing planned work due to cellphone use” and “experiencing difficulty concentrating in class, completing homework, or while at work because of smartphone usage.” Scores on a 5-point Likert scale (1 = strongly disagree to 5 = strongly agree) are used to determine the level of smartphone addiction, where higher scores suggest a greater level of smartphone addiction.

### 2.3 Statistical analyses plan

SPSS 27.0, Process macro and AMOS 24 were used to analyze the data. First, descriptive statistics, normality, and correlations were examined with SPSS 27.0. Second, AMOS was used to test the direct effects of isolation, loneliness, and COVID-19 anxiety on smartphone addiction. Finally, the mediating effect was assessed with the PROCESS (version 4.1) macro model 6. In addition, reverse mediator models of loneliness and COVID-19 anxiety were tested.

## 3 Data analysis

### 3.1 Preliminary analyses

The demographic profiles of the respondents are listed in Table 2. The majority of the participants (*n* = 849, 97.8%) were 18–25 years of age. The ratio of females (*n* = 620, 71.4%) to males (*n* = 248, 28.6%) was relatively similar to the ratios in the eight higher education institutions in which the students were enrolled. Participants were enrolled in junior (*n* = 291, 33.5%), sophomore (*n* = 256, 29.5%), or senior (*n* = 224, 11.2%) grades. Living expenses were concentrated at 1000–1500 (*n* = 338, 38.9%) and 1,500–2000 (*n* = 263, 30.3%).

**Table 2:** Demographic profiles of the respondents (*N* = 868).

Variables	Items	Frequency	Percent (%)	Mean of AD	*p*-value
Gender^a^	Male	248	28.6	3.80	*p* > 0.05
	Female	620	71.4	3.77	
Age^b^	Below 18	19	2.2	4.06	*p* > 0.05
	18–25	849	97.8	3.77	
Monthly living expenses	Below CNY 1000^c^	67	7.7	3.99	*p* < 0.05
	CNY 1000–1500	338	38.9	3.73	
	CNY 1500–2000	263	30.3	3.67	
	CNY 2000–2,500	125	14.4	3.95	
	Above CNY 2500	75	8.6	3.90	
College	Guangdong Engineering Polytechnic	80	9.2	4.06	*p* < 0.01
	Guangzhou Medical College Institute of Conghua	60	6.9	3.68	
	Nanfang College · Guangzhou	158	18.2	3.54	
	Guangdong Polytechnic of Water Resources and Electric Engineering	141	16.2	3.90	
	Zhujiang College of South China Agricultural University	80	9.2	3.77	
	Software Engineering Institute of Guangzhou	102	11.8	3.83	
	Guangzhou Nanyang Polytechnic College	92	10.6	3.75	
	Guangzhou City Construction College	155	17.9	3.80	
Grade	Freshman	97	11.2	3.84	*p* < 0.01
	Sophomore	256	29.5	3.80	
	Junior	291	33.5	3.62	
	Senior	224	25.8	3.94	
Total		868	100		

^a^and ^b^use independent sample *T*-test analysis.

c CNY, Chinese Yuan. 1 CNY = 7.2 dollars. AD = smartphone addiction.

Table 3 displays the descriptive statistics and correlations between the focal variables. The distribution of the four focus variables was normal, with skewness and kurtosis varying from −2 to 2 (Curran et al., 1996). This suggested that subsequent data analysis could be conducted. In addition, Table 3 shows the bivariate correlations among the focal variables, indicating significant correlations among social isolation, loneliness, COVID-19 anxiety, and smartphone addiction, ranging from 0.583 to 0.69, *p* < 0.01.

**Table 3:** Descriptive statistic, reliability, and correlations among variables (*N* = 868).

Variable	M	SD	Skewness	Kurtosis	1	2	3	4
1. IS	3.862	0.888	−1.717	1.932	–			
2. LO	3.762	1.017	−1.178	0.234	0.606**	–		
3. AN	3.744	0.938	−1.233	0.565	0.607**	0.583**	–	
4. AD	3.780	0.939	−1.311	0.659	0.630**	0.631**	0.690**	–

All tests are two-tailed. M, mean; SD, standard deviation; IS, social isolation; LO, loneliness; AN, COVID-19 anxiety; AD, smartphone addiction. *p < 0.05, **p < 0.01, ***p < 0.001.

### 3.2 Variance analysis of demographic factors on smartphone addiction

This study revealed that the college student population in Conghua District exhibited a high level of smartphone addiction (mean = 3.78) at the end of 2022, in line with the results of previous studies (Santander-Hernández et al., 2022; Li et al., 2023) (Table 3). The results of the one-way ANOVA and *T*-test indicated that college (F = 2.937, *p* ≤ 0.01), monthly living expenses (F = 3.277, *p* ≤ 0.05), and grade (F = 5.059, *p* ≤ 0.01) on smartphone addiction are significant differences, while gender and age on smartphone addiction are insignificant differences (Table 2). More specifically, the students at Guangdong Engineering Polytechnic have the highest level of smartphone addiction (mean = 4.06), followed by Guangdong Polytechnic of Water Resources (mean = 3.90) and Electric Engineering and Software Engineering Institute of Guangzhou (mean = 3.83). Among the eight higher education institutions in Conghua, students from science and engineering colleges may have significantly higher levels of cellphone addiction than those from other comprehensive colleges. Besides, senior students (mean = 3.94) and freshman (mean = 3.84) have a higher degree of smartphone addiction than sophomore and junior students. Students whose monthly living expenses are below 1,000 (RMB) hold the highest level of smartphone addiction (mean = 3.99), followed by students whose monthly living expenses are between 2,000 and 2,500 (RMB) (mean = 3.96).

### 3.3 Examination of the structural equation model

#### 3.3.1 Measurement model

Initially, the reliability of the constructs was evaluated through Cronbach’s alpha, confirming that all the constructs exceeded the cutoff value of 0.7 (Nunnally, 1978) (Table 4). The results of confirmatory factor analysis (CFA) indicated an excellent model fit, as all indices met the recommended values: χ2 (246) =507.875, χ2/df = 2.605 (<3), root mean square error of approximation (RMSEA) = 0.035 (<0.05), comparative fit index (CFI) = 0.985 (>0.9), Tucker–Lewis index (TLI) = 0.984 (>0.9), and standardized root mean square residual (SRMR) = 0.023 (<0.08).

**Table 4:** Reliability, factor loading and convergent validity (*N* = 868).

Variables	Items	Cronbach’s alpha	Factor loading	CR	AVE
IS	IS1	0.913	0.787	0.914	0.639
	IS2		0.789		
	IS3		0.79		
	IS4		0.811		
	IS5		0.824		
	IS6		0.796		
LO	LO1	0.886	0.847	0.887	0.724
	LO2		0.838		
	LO3		0.867		
AN	AN1	0.916	0.824	0.918	0.692
	AN2		0.84		
	AN3		0.841		
	AN4		0.854		
	AN5		0.798		
AD	AD1	0.963	0.837	0.963	0.725
	AD2		0.863		
	AD3		0.803		
	AD4		0.882		
	AD5		0.849		
	AD6		0.818		
	AD7		0.88		
	AD8		0.858		
	AD9		0.865		
	AD10		0.858		

IS, isolation; LO, loneliness; AN, COVID-19 anxiety; AD, smartphone addiction.

Subsequently, convergent validity and discriminant validity tests were conducted to examine the construct validity of the latent variables (Table 4). Convergent validity was evaluated by the average variance extracted (AVE) value and composite reliability (CR). The value of the AVE ranged from 0.639 to 0.725, higher than the cutoff value of 0.5 (Fornell and Larcker, 1981). The CR values for the variables were all greater than the recommended value of 0.7.

By comparing the square root of the AVE values with the absolute values of the correlation coefficients between the variables, discriminant validity was assessed (Fornell and Larcker, 1981). Table 5 shows that all the AVE values’ square roots were larger than the correlation coefficients’ absolute values between the relevant latent variables and other factors. Consequently, the discriminant validity was deemed excellent. In conclusion, the CFA findings demonstrated the validity and reliability of the measurement model (Schmitt, 2012).

**Table 5:** Discriminate validity (*N* = 868).

	AVE	IS	LO	AN	AD
IS	0.638	**0.799**			
LO	0.724	0.674	**0.851**		
AN	0.692	0.662	0.646	**0.832**	
AD	0.725	0.676	0.687	0.737	**0.852**

IS, isolation; LO, loneliness; AN, COVID-19 anxiety; AD, smartphone addiction.

#### 3.3.2 Structural model testing

The results of structural model testing indicated a good model fit: χ2(246) = 507.875, χ2/df = 2.065, RMSEA = 0.035, CFI = 0.951, TLI = 0.939, and SRMR = 0.023. After determining the relative goodness of fit of the model, the next step was to assess the individual path coefficients according to our hypotheses.

The results of the path analysis are shown in Table 6. Social isolation (β = 0.215, *p* < 0.001), loneliness (β = 0.271, *p* < 0.001), and COVID-19 anxiety (β = 0.42, *p* < 0.001) were positively associated with smartphone addiction, supporting H1, H3, and H6. In addition, social isolation (β = 0.416, *p* < 0.001) and loneliness (β = 0.365, *p* < 0.001) were positively related to COVID-19 anxiety, supporting H5 and H8. Finally, social isolation (β = 0.674, *p* < 0.001) was significantly positively associated with loneliness, supporting H2.

**Table 6:** Path analysis (*N* = 868).

Hypotheses	Path	β	*t* value	Support
H1	IS → AD	0.215	5.68***	Positive
H2	IS→ LO	0.674	18.048***	Positive
H3	LO → AD	0.271	7.142***	Positive
H5	IS→ AN	0.416	9.674***	Positive
H6	AN→ AD	0.42	11.11***	Positive
H8	LO → AN	0.365	8.578***	Positive

*p < 0.05, **p < 0.01, ***p < 0.001. IS, isolation; LO, loneliness; AN, COVID-19 anxiety; AD, smartphone addiction.

### 3.4 Examination of multiple mediating effects

**Figure 2:**
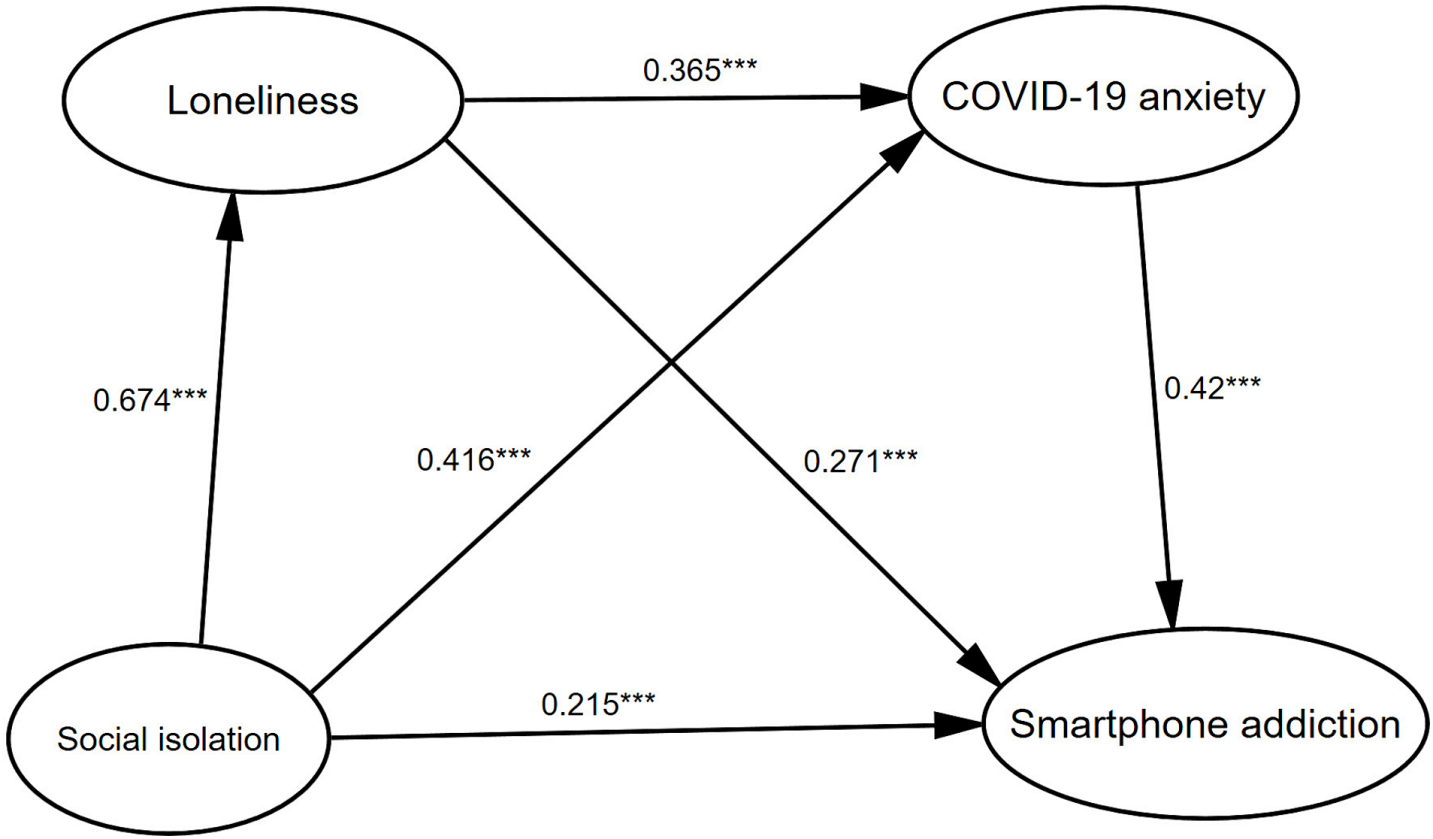
The multiple mediation model of social isolation on smartphone addiction, with loneliness as the first mediator and COVID-19 anxiety as the second (*N* = 868). Standard coefficients are reported. All the path coefficients were significant. *p < 0.05, **p < 0.01, ***p < 0.001.

To examine the link between social isolation and smartphone addiction, a mediation analysis was carried out using loneliness and COVID-19 anxiety as mediators through the Hayes PROCESS model 6 (Table 7). This analysis employed ordinary least squares path analysis for more precise estimation by bootstrapping with 5,000 samples (Igartua and Hayes, 2021). The mediating effects of loneliness (β = 0.165, SE = 0.024, *p* < 0.001, 95% CI [0.213, 0.248]) and COVID-19 anxiety (β = 0.171, SE = 0.023, *p* < 0.001, 95% CI [0.217, 0.257]) were significant, accounting for 24.8 and 25.7% of the total effect, respectively, which supported H4 and H7. Even with the inclusion of two mediators, the direct impact of social isolation on smartphone addiction remained significant (β = 0.243, SE = 0.032, *p* < 0.001, 95% CI [0.305, 0.365]). The mediating effect of loneliness and COVID-19 anxiety in sequence was also significant (β = 0.087, SE = 0.015, 95% CI [0.059, 0.119]), supporting H9 (Figure 2; Table 7).

**Table 7:** Bootstrapping analysis of the mediating effects (*N* = 868).

	Mediation model					Reverse mediation model				
	β	SE	LLCI	ULCI	Ratio	β	SE	LLCI	ULCI	Ratio
Total effect	0.665	0.028	0.610	0.721	–	0.665	0.028	0.610	0.721	–
Direct effect	0.243	0.032	0.180	0.305	36.5%^a^	0.243	0.032	0.180	0.305	36.5%^a^
Indirect total effect	0.423	0.028	0.366	0.477	63.5%^a^	0.423	0.028	0.366	0.477	63.6%^a^
IS - > LO - > AD	0.165	0.024	0.120	0.213	24.8%^b^	0.109	0.017	0.077	0.144	16.4%^b^
IS - > AN - > AD	0.171	0.023	0.126	0.217	25.7%^b^	0.256	0.028	0.203	0.311	38.6%^b^
IS - > LO - > AN - > AD	0.087	0.015	0.059	0.119	13.1%^b^	–				
IS - > AN - > LO - > AD	–					0.057	0.012	0.035	0.083	8.5%^b^

a Proportion of total effect.

b Proportion of indirect total effect.

IS, isolation; LO, loneliness; AN, COVID-19 anxiety; AD, smartphone addiction.

**Figure 3:**
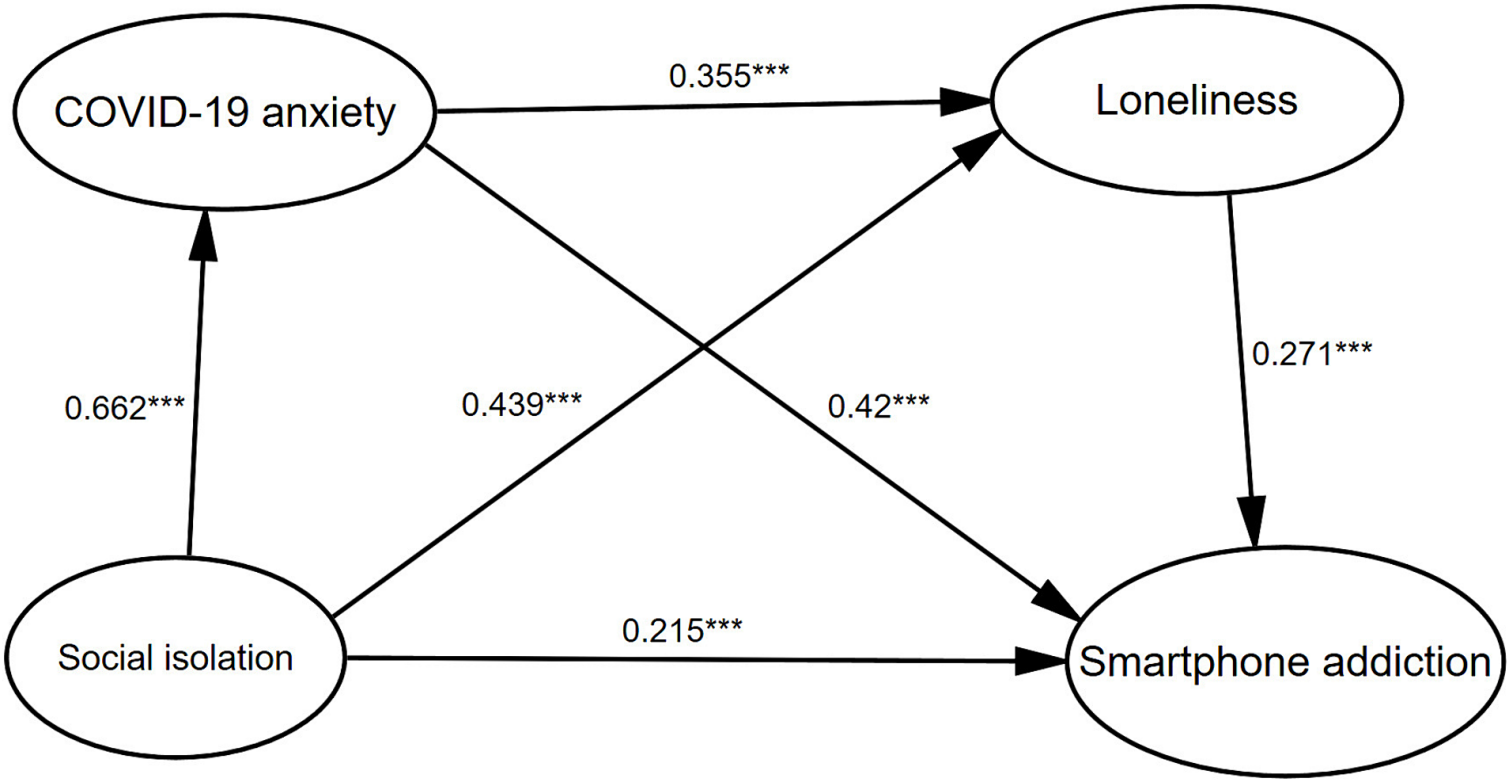
The multiple mediation model of social isolation and smartphone addiction, with COVID-19 anxiety as the first mediator and loneliness as the second mediator (*N* = 868). Standard coefficients are reported. All the path coefficients are significant. *p < 0.05, **p < 0.01, ***p < 0.001.

In the exploratory reverse mediation model (Figure 3; Table 7), the direct influences of social isolation, loneliness, and COVID-19 anxiety on smartphone addiction mirrored those observed in the initial mediation model. COVID-19 anxiety (individually mediated β = 0.256, SE = 0.028, 95% CI [0.203, 0.311]) and loneliness (individually mediated β = 0.109, SE = 0.017, 95% CI [0.077, 0.144]) serially mediated (β = 0.057, SE = 0.012, 95% CI [0.035, 0.083]) the relationship between social isolation and smartphone addiction.

## 4 Discussion

This study constructed a multiple mediation model to clarify how social isolation was associated with smartphone addiction among college students from Conghua District, Guangzhou city, China. Specifically, the results illustrated that social isolation could positively predict loneliness and COVID-19 anxiety, and in turn, loneliness and COVID-19 anxiety were positively associated with smartphone addiction. Furthermore, loneliness could also lead to increased COVID-19 anxiety, which showed a sequential mediation. The following paragraph will discuss each of the research hypotheses in light of this multiple mediation model.

This study demonstrated the direct effects of social isolation, loneliness, and COVID-19 anxiety on smartphone addiction (H1, H3, H6). Besides, loneliness and COVID-19 anxiety mediated the relationship between social isolation and smartphone addiction (H4, H7). Furthermore, loneliness and COVID-19 anxiety mediated the association between social isolation and smartphone addiction in both parallel and sequential way (H9). Moreover, a reverse mediation model was identified, in which COVID-19 anxiety was the first mediator and loneliness was the second mediator. In conclusion, these results confirm our hypothesis to a certain extent and clarify the potential mechanisms underlying the relationship between social isolation and smartphone addiction in the post-pandemic era.

This study confirmed that the direct effect of social isolation on smartphone addiction was significant, which is consistent with previous findings (e.g., Al-Kandari and Al-Sejari, 2021; Brailovskaia and Margraf, 2021; Zhen et al., 2023). Our study confirmed that people who experienced social isolation were more susceptible to smartphone addiction during the COVID-19 pandemic. In this study, social isolation was considered a composite dimension that included both objective states and subjective feelings (Leal Filho et al., 2021), thus enriching the literature related to social isolation in the context of infectious diseases, especially in the late stage of COVID-19.

More importantly, this study demonstrated the mediating roles of loneliness and COVID-19 anxiety on smartphone addiction. Usage and gratification theory and CIU theory suggest that smartphone addiction in college students is a positive response to stressful events such as public health emergencies in an attempt to meet psychological needs, although it may lead to negative outcomes (e.g., addiction). People use smartphones for a variety of purposes (Katz et al., 1974), including information retrieval, facilitating social engagement, alleviating boredom, providing distractions, mitigating negative feelings, and enhancing positive emotions (Brailovskaia et al., 2020). Notably, loneliness mediated the relationship between social isolation and smartphone addiction. This pathway is based on the motivation for the emotional use of smartphones, which creates a sense of loneliness when college students are socially isolated for a long period, and smartphones not only help individuals engage in online social interactions and establish virtual social relationships but also complement offline social relationships and satisfy users’ social needs. Individuals with high levels of loneliness are prone to develop cell phone addiction (Shen and Wang, 2019). In addition, COVID-19 anxiety mediated the relationship between social isolation and smartphone addiction. Social isolation triggered more concerns about COVID-19, especially in late 2022, when old and new closure policies were in place, coinciding with the winter and Chinese New Year holidays, prolonged social isolation coupled with a desire to return home increased concerns, and college students used their smartphones to search for information related to COVID-19 (e.g., government policies, COVID-19 trends, preventive measures, information on vaccines and medications) or to engage in online entertainment and pastimes to alleviate anxiety, which may have led to smartphone addiction.

Notably, the analysis of the mediating effect indicated (Figures 2, 3) that the impact of loneliness on COVID-19 anxiety may be reciprocal. Empirical studies have confirmed the impact of loneliness on COVID-19 anxiety; however, there is very little evidence on the impact of COVID-19 anxiety on loneliness (e.g., Wu et al., 2022). On the one hand, lonely individuals usually receive less social support (Wang et al., 2018), such as emotional support and information support, because of the lack of timely updates on COVID-19 pandemic information and social–emotional support, which undoubtedly exacerbates COVID-19 anxiety (Al-Kandari and Al-Sejari, 2021). COVID-19 anxiety is different from loneliness in that it leads people to worry about future events, whereas loneliness is a state of emotional discomfort that is focused on the present. In this sense, loneliness may increase the salience of anxiety because individuals lack relevant coping (e.g., social gatherings, seeking peer advice) to alleviate loneliness (Hoffart et al., 2020), thereby exacerbating the risk of smartphone addiction. On the other hand, anxiety and worry may increase the intensity of loneliness, as they may amplify the negative emotions that people fear will continue in the future (Mayorga et al., 2022). Therefore, individuals first develop symptoms of COVID-19 in response to social isolation anxiety, which in turn exacerbates feelings of loneliness and ultimately leads to smartphone addiction. Because of our cross-sectional design, the results should be interpreted as a guide to exploring the relationship rather than a basis for determining the causal relationships between them. The relationship between anxiety and loneliness during the COVID-19 pandemic may be correlational, predictive, or reciprocal. Future research should use cross-lagged analyses to reveal the directionality of the relationship between loneliness and COVID-19 anxiety. Although the sequence of the two mediators remains indeterminate, our findings could pave the way for innovative strategies for preventing, identifying, and intervening in smartphone addiction.

Finally, the indirect path between social isolation and smartphone addiction was more significant (β = 0.423, ratio = 63.5%) than the direct path (β = 0.243, ratio = 36.5%). This reveals that the core factors leading to smartphone addiction are subjective psychological states, such as the loneliness and anxiety triggered by campus isolation and social distancing. Social isolation has a significant impact on the mental health of undergraduates (Mayorga et al., 2022). Therefore, it is important to continue to focus on the mental health of college students even after COVID-19 has ended. The implementation of effective psychological interventions may reduce the loneliness and anxiety experienced by students during the period of enforced isolation due to COVID-19, thereby reducing smartphone addiction. It is recommended that colleges and universities establish professional psychological counseling services and implement various forms of mental health education activities to correct and prevent smartphone addiction among college students. Comprehensive support from schools, families, and communities can improve students’ social interaction skills (Al-Kandari and Al-Sejari, 2021), meet their diverse social and emotional needs, and reduce their dependence on smartphones. In addition, comprehensive and multidimensional information about COVID-19-related dynamics and adequate informational support can further reduce COVID-19 anxiety.

## 5 Conclusion

This study explored the potential mechanisms underlying the relationship between social isolation and smartphone addiction. Social isolation not only directly predicted smartphone addiction but also predicted smartphone addiction through loneliness and COVID-19 anxiety, both in a parallel and sequential way. Moreover, an unexpected reverse mediation model with COVID-19 anxiety as the first mediator and loneliness as the second mediator was found. The results of the study suggest that college students experiencing social isolation are at greater risk for smartphone addiction, and the core factor leading to their smartphone addiction is the subjective psychological state triggered by campus isolation and social distancing, such as loneliness and anxiety. This study enriches our understanding of why and how college students experiencing social isolation exhibit smartphone addiction. The findings also extend the theory of antecedent research on cell phone addiction in the context of public health crises. This study also provides a theoretical basis and practical guidance for addressing smartphone addiction among college students in the context of public health crises.

## 6 Limitations

This study has several limitations. The adoption of a cross-sectional survey method precludes the determination of causal links among the studied variables. Subsequent inquiries are encouraged to employ longitudinal or experimental methodologies to rigorously examine this multi-mediation model. Second, only some college students in Conghua District, Guangzhou city, were included, and the quota sample was a nonprobability sample. Although nonprobability samples are acceptable for studies aimed at testing and developing theoretical models of multiple relationships (Basil et al., 2002), the findings cannot be generalized, and future work should use probability samples to increase the generalizability of the conclusions. Third, self-report questionnaires may lead to measurement bias due to social desirability. Moreover, respondents tend to underestimate their own situations and minimize their certain behavioral problems related to smartphone addiction (Wu-Ouyang and Chan, 2023); therefore, the data obtained often do not reflect the real situation (Brailovskaia and Margraf, 2021). Future research should apply the smart experience sampling method (Otto and Kruikemeier, 2023) to accurately measure the behavior and psychology of participants. Fourth, there is a body of literature suggesting that loneliness, anxiety, and smartphone addiction have bidirectional effects. Specifically, loneliness and anxiety can trigger smartphone addiction, and smartphone addiction further increases loneliness and anxiety (e.g., Demirci et al., 2015; Santander-Hernández et al., 2022). Future research should further investigate this bidirectional relationship. Finally, while this research focused on the link between social isolation and smartphone addiction in college students, it overlooked additional variables such as social support (Hou et al., 2023) or sense of control (Brailovskaia and Margraf, 2021). Future inquiries should incorporate these elements to achieve a more holistic understanding of the factors contributing to smartphone addiction.

## 7 Contributions

Despite its limitations, this study has several implications. Theoretically, this study pioneers the investigation of the dynamic interplay between social isolation, loneliness, COVID-19 anxiety, and smartphone addiction; uniquely reveals both the parallel and sequential mediating roles of loneliness and COVID-19 anxiety; and uncovers bidirectional correlations between loneliness and COVID-19 anxiety. This study adds significantly to our knowledge of smartphone addiction and to the body of literature on the causes of smartphone addiction in public crisis situations.

Practically, this study focuses on the transition between old and new containment policies for the COVID-19 pandemic while expanding the research perspective to include the public crisis caused by the pandemic, the quarantine requirements of the policy, and the psychological problems of college students triggered by the specific context of closed university management and exploring the antecedents of smartphone addiction in terms of psychological compensation mechanisms. Although at the time of writing, the Chinese government announced that COVID-19 had ended and that the quarantine policy had been relieved, situations and challenges may recur during future epidemics, with relatively stable influences and internal patterns behind them. Moreover, this study included college students in Conghua District (Guangzhou city), which has a strong geographical character. Therefore, this study is important for formulating preventive and interventional approaches to address smartphone addiction among college students during infectious disease outbreaks.

## Data availability statement

The original contributions presented in the study are included in the article/Supplementary material, further inquiries can be directed to the corresponding author.

## Ethics statement

The studies involving humans were approved by the Academic Ethics Committee of Nanfang College · Guangzhou. The studies were conducted in accordance with the local legislation and institutional requirements. Written informed consent for participation was not required from the participants or the participants' legal guardians/next of kin in accordance with the local legislation and institutional requirements. However, the research design was rigorously reviewed to ensure anonymity and voluntary participation.

## Author contributions

YW: Conceptualization, Investigation, Project administration, Resources, Supervision, Writing – original draft, Writing – review & editing, Validation. QM: Data curation, Funding acquisition, Methodology, Software, Visualization, Writing – original draft, Writing – review & editing, Formal analysis.
