# Supplementary material for: The impact of social isolation on smartphone addiction among college students: the multiple mediating effects of loneliness and COVID-19 anxiety
Source: Front Psychol. 2024 Jul 22;15:1391415. doi: 10.3389/fpsyg.2024.1391415 (PMC11299513; doi:10.3389/fpsyg.2024.1391415)
Supplement: Supplementary file 1 [file Data_Sheet_1.docx]

Measurement of focal variables

**Social isolation scale**

| 6. Among your family, friends, or neighbors, how many people do you see at least once a month? | | | | |
| --- | --- | --- | --- | --- |
| □A.6 and more | □B.4-5 pieces | □C.2-3 pieces | □D.1 | □E.No |
| 7. Among your family, friends, or neighbors, how many people do you communicate with at least once a month by phone, electronic means (such as email, video chat), or the internet? | | | | |
| □A.6 and more | □B.4-5 pieces | □C.2-3 pieces | □D.1 | □E.No |
| 8. Among your family, friends, or neighbors, how many people do you feel personally close to (for example, you can confide in them or share personal feelings with)? | | | | |
| □A.6 and more | □B.4-5 pieces | □C.2-3 pieces | □D.1 | □E.No |
| 9. In general, you feel that your relationships with individuals or groups to which you belong are not fulfilling. | | | | |
| □A.Strongly disagree | □B. I slightly disagree | □C.Neither agree nor disagree | □D. Somewhat agree | □E.Strongly agree |
| 10. You feel that you do not belong in the personal or group relationships to which you belong. | | | | |
| □A.Strongly disagree | □B. I slightly disagree | □C.Neither agree nor disagree | □D. Somewhat agree | □E.Strongly agree |
| 11. In your relationship with an individual or group to which you belong, you feel that you do not spend enough time participating in social activities | | | | |
| □A.Strongly disagree | □B. I slightly disagree | □C.Neither agree nor disagree | □D. Somewhat agree | □E.Strongly agree |

**Loneliness scale**

| 1 2. How often do you feel that you lack company? | | | | |
| --- | --- | --- | --- | --- |
| □A.Never | □B. Very few | □C.Sometimes | □D. often | □E.Always |
| 1 3. How many times have you felt left out? | | | | |
| □A.Never | □B. Very few | □C.Sometimes | □D. often | □E.Always |
| 14. Do you often feel isolated from others? | | | | |
| □A.Never | □B. Very few | □C.Sometimes | □D. often | □E.Always |

**COVID-19 Anxiety Scale**

| 15. You feel anxious about your future because of the COVID-19 epidemic . | | | | |
| --- | --- | --- | --- | --- |
| □A.Strongly disagree | □B. I slightly disagree | □C.Neither agree nor disagree | □D. Somewhat agree | □E.Strongly agree |
| 16. You are worried about your health because of COVID- 19 | | | | |
| □A.Strongly disagree | □B. I slightly disagree | □C.Neither agree nor disagree | □D. Somewhat agree | □E.Strongly agree |
| 17. Are you anxious about being infected with the new coronavirus? | | | | |
| □A.Strongly disagree | □B. I slightly disagree | □C.Neither agree nor disagree | □D. Somewhat agree | □E.Strongly agree |
| 18. You are worried about being infected with COVID-19. | | | | |
| □A.Strongly disagree | □B. I slightly disagree | □C.Neither agree nor disagree | □D. Somewhat agree | □E.Strongly agree |
| 19. Are you worried that the COVID-19 epidemic will affect your life? | | | | |
| □A.Strongly disagree | □B. I slightly disagree | □C.Neither agree nor disagree | □D. Somewhat agree | □E.Strongly agree |

**Smartphone Addiction Scale**

| 20. Missing planned work due to smartphone use | | | | |
| --- | --- | --- | --- | --- |
| □A.Strongly disagree | □B. I slightly disagree | □C.Neither agree nor disagree | □D. Somewhat agree | □E.Strongly agree |
| 21. Difficulty concentrating in class, doing homework or working due to the use of smartphones | | | | |
| □A.Strongly disagree | □B. I slightly disagree | □C.Neither agree nor disagree | □D. Somewhat agree | □E.Strongly agree |
| 22. Feeling pain in the wrist or back of the neck when using a smartphone | | | | |
| □A.Strongly disagree | □B. I slightly disagree | □C.Neither agree nor disagree | □D. Somewhat agree | □E.Strongly agree |
| 23. Will not be able to bear the days without smartphones | | | | |
| □A.Strongly disagree | □B. I slightly disagree | □C.Neither agree nor disagree | □D. Somewhat agree | □E.Strongly agree |
| 24. Feeling impatient and irritable when you are not holding your smartphone | | | | |
| □A.Strongly disagree | □B. I slightly disagree | □C.Neither agree nor disagree | □D. Somewhat agree | □E.Strongly agree |
| 25. Even when you are not using your smartphone, your phone will appear in your mind | | | | |
| □A.Strongly disagree | □B. I slightly disagree | □C.Neither agree nor disagree | □D. Somewhat agree | □E.Strongly agree |
| 26. You will never give up using your smartphone, even if your daily life has been greatly affected by it | | | | |
| □A.Strongly disagree | □B. I slightly disagree | □C.Neither agree nor disagree | □D. Somewhat agree | □E.Strongly agree |
| 27. Keep checking your smartphone so you don’t miss conversations with other people. | | | | |
| □A.Strongly disagree | □B. I slightly disagree | □C.Neither agree nor disagree | □D. Somewhat agree | □E.Strongly agree |
| 28. Use your smartphone for longer than you expect | | | | |
| □A.Strongly disagree | □B. I slightly disagree | □C.Neither agree nor disagree | □D. Somewhat agree | □E.Strongly agree |
| 29. People around you tell you that you use your smartphone too much | | | | |
| □A.Strongly disagree | □B. I slightly disagree | □C.Neither agree nor disagree | □D. Somewhat agree | □E.Strongly agree |
